# Miscellaneous

**Published:** 1884-03

**Authors:** 


### Miscellaneous.

### OFFICIAL LIST OF CHANGES OF STATIONS AND DUTIES OF MEDICAL OFFICERS OF THE UNITED STAES MARINE HOSPITAL SERVICE, OCTOBER 1, 1883, TO DECEMBER 31, 1883.

Bailhache, P. H., Surgeon, relieved from duty at Cape Charles Quarantine Station, Oct. 13, 1883.

Detailed as member of Board to examine candidates for promotion Oct. 30, 1883.

Granted leave of absence for thirty days, Nov. 27, 1883.

Hutton, W. H. H., Surgeon, granted leave of absence for twenty days, Oct. 1, 1883.

Wyman, Walter, Surgeon, detailed as member of Board to examine candidates for promotion, Oct. 30, 1883.

To proceed to Norfolk, Va., to investigate the conduct of the service at that port, Dec. 31, 1883.

Long, W. H., Surgeon, leave of absence extended ten days, Oct. 26, 1883.

Murray, R. D., Surgeon, to proceed to Ship Island Quarantine Station, Oct. 17, 1883.

To inspect sites for quarantine stations, Nov. 30, 1883.

Granted leave of absence for twenty days, Dec. 18, 1883.

Smith, Henry, Surgeon, granted leave of absence for twenty-five days on account of sickness, Oct. 13, 1883.

Relieved from duty at Norfolk, Va., Oct. 17, 1883.

To report to Surgeon Sawtelle, at New York, for temporary duty, Nov. 27, 1883.

Relieved from temporary duty at New York, and placed on waiting orders, Dec. 31, 1883.

Fisher, J. C., Passed Assistant Surgeon, when relieved by Assistant Surgeon Banks, to proceed to New York for duty, Oct. 29, 1883.

Granted leave of absence for thirty days, Nov. 28, 1883.

Goldsborough, C. B., Passed Assistant Surgeon, granted leave of absence for thirty-two days, on account of sickness, Oct. 12, Oct. 20, and Nov. 1, 1883.

Irvin, Fairfax, Passed Assistant Surgeon, to proceed to Norfolk, Va., and assume charge of the service, relieving Assistant Surgeon Glennan, Oct. 16, 1883.

Mead, F. W. Passed Assistant Surgeon, to proceed to Portland, Or., inspect the service, and report the condition of Assistant Surgeon Devan, Dec. 5, 1883.
To return to station, Port Townsend, Wash. Ter., Dec. 18, 1883.

Cooke, H. P., Passed Assistant Surgeon, to proceed to Charleston, S.C., for duty, Nov. 27, 1883.

Banks, C. E., Assistant Surgeon, detailed for temporary duty at Georgetown, D.C., Oct. 11, 1883.
Granted leave of absence for thirty days, Oct. 12, 1883.

Bennett, P. H., Assistant Surgeon, placed on waiting orders, Dec. 15, 1883.
Granted leave of absence for thirty days, Dec. 22, 1883.
Upon expiration of leave of absence to proceed to Detroit, Mich., for duty, Dec. 29, 1883.

Peckham, C. T., Assistant Surgeon, to proceed to Wilmington, N. C., and assume charge of the Service, relieving Passed Assistant Surgeon Irwin, Oct. 16, 1883.

Devan, S. C., Assistant Surgeon, granted leave of absence for ninety-five days, on account of injury and sickness resulting therefrom, Nov. 15, Dec. 5 and 22, 1883.

Bevan, A. D., Assistant Surgeon, to proceed to Portland, Or., and assume charge of the Service, Dec. 29, 1883.

Glennan, A. H., Assistant Surgeon, to proceed to New Orleans, La., for duty Oct, 17, 1883.

Wasdin, Eugene, Assistant Surgeon. to proceed to Mobile, Ala., for temporary duty, Oct. 11, 1883.
To proceed to Galveston, Texas, for temporary duty, Nov. 17, 1883.

PROMOTIONS.

Benson, J. A., Passed Assistant Surgeon, promoted and appointed Passed Assistant Surgeon, by the Secretary of the Treasury, from Oct. 1, 1883. Oct. 4, 1883.

Banks, C. E., Passed Assistant Surgeon, promoted and appointed Passed Assistant Surgeon, by the Secretary of the Treasury, from Nov. 1, 1883. Nov. 6, 1883.

COLLEGE OF PHYSICIANS OF PHILADELPHIA.—The following officers were elected at the annual meeting of the College of Physicians of Philadelphia, held Jan. 2 : President, Dr. Samuel Lewis; Vice-President, Dr. J. M. DaCosta; Secretary, Dr. Richard A. Cleeman; Treasurer, Dr. Charles S. Wurst.—*Mary-and Medical Journal.*

### OFFICIAL LIST OF MEDICAL OFFICERS AND ACTING ASSISTANT SURGEONS OF THE UNITED STATES MARINE-HOSPITAL SERVICE, WITH THEIR STATIONS. JANUARY, 1884.

MEDICAL OFFICERS.

| NAME AND RANK. | STATION. | NAME AND RANK. | STATION. |
|---|---|---|---|
| *Supervising Surgeon-General.* | | F. W. Mead | Port Townsend, W. T, |
| John B. Hamilton | Washington, D. C. | H. P. Cooke | Charleston, S. C. |
| *Surgeons.* | | H. R. Carter | San Francisco, Cal. |
| P. H. Bailhache | Washington, D. C. | W. H. Heath | Buffalo, N. Y. |
| John Vansant | San Francisco, Cal. | John Guitéras | Key West, Fla. |
| W. H. H. Hutton | Louisville, Ky. | W. A. Wheeler | Chicago, Ill. |
| T. W. Miller | Chicago, Ill. | J. A. Benson | Cairo, Ill. |
| Walter Wyman | Baltimore, Md. | C. E. Banks | Washington, D. C. |
| W. H. Long | Detroit, Mich. | *Assistant Surgeons.* | |
| R. D. Murray | Gulf Quarantine. | D. A. Carmichael | Pittsburgh, Pa. |
| C. S. D Fessenden | St. Louis, Mo. | S. T. Armstrong | Memphis, Tenn. |
| Geo. Purviance | Boston, Mass. | P. H. Bennett | Detroit, Mich. |
| H. W. Sawtelle | New York, N. Y. | C. T. Peckham | Wilmington, N. C. |
| H. W. Austin | Cincinnati, Ohio. | R. P M. Ames | Evansville, Ind. |
| J. M. Gassaway | Philadelphia, Pa. | S C Devan† | |
| Henry Smith* | | F. M. Urquhart | St. Louis, Mo. |
| *Passed Ass'ant Surgeons.* | | P. C. Kalloch | New York, N. Y. |
| G. W. Stoner | Portland, Me. | H. W. Yemans | San Francisco, Cal. |
| J. C. Fisher | New York, N. Y. | A. D. Bevan | Portland, Oregon. |
| John Godfrey | New Orleans, La. | A. H. Glennan | New Orleans, La. |
| C. B. Goldsborough | Mobile, Ala. | Eugene Wasdin‡ | Galveston, Texas. |
| Fairfax Irwin | Norfolk, Va. | *Surgeon,* (Retired). | |
| | | T. J. Griffiths§ | Louisville, Ky. |

*On waiting orders. †On sick leave. ‡ Temporary. § Consulting Surgeon, Louisville Marine Hospital.

ACTING ASSISTANT SURGEONS.

| NAME. | STATION. | NAME. | STATION. |
|---|---|---|---|
| J. M. Allen | Milwaukee, Wis. | J. M. Main | Brownsville, Texas. |
| W. A. Banks | Rockland, Me. | W. C. Mason | Bangor, Me. |
| H. G. Bates | New Berne, N. C. | H. E. Mereness | Albany, N. Y. |
| B. F. Beebe | Cincinnati, Ohio. | J. D. Mitchell | Jacksonville, Fla. |
| R. D. Bibber | Bath, Me. | P. H. C. Noble | Richmond, Va. |
| J. E. Bready | Dubuque, Iowa. | Chas. Ottilie | La Crosse, Wis. |
| J. B. Brewster | Plymouth, Mass. | W. R. Page | Sandusky, Ohio. |
| S. D. Brooks | Boston, Mass. | T. T. Price | Tuckerton, N. J. |
| G. B Case | Cleveland, Ohio. | S. D. Robbins | Vicksburg, Miss. |
| S. B. Conover | Philadelphia, Pa. | J. A. Rowles | Chicago, Ill. |
| W. A. Cox | Pascagoula, Miss. | S. H. Sears | Newport, R. I. |
| J. B. Cromley | Gallipolis, Ohio. | Elmer Small | Belfast, Me. |
| Byron DeWitt | Oswego, N. Y. | W N. Smart | Grand Haven, Mich. |
| Richard Douglas | Nashville, Tenn. | A. E Spohn | Corpus Christi, Texas. |
| A. W. Fisher | Toledo, Ohio. | J. G. Stanton | New London, Conn. |
| J. P. C. Foster | New Haven, Conn. | Theodore Starbuck | Fernandina, Fla. |
| T. L. Gelzer | Escanaba, Mich. | W. D. Stewart | Vineyard Haven, Mass. |
| L. P. Gibson | Little Rock, Ark. | G. H. Stone | Savannah, Ga. |
| Thos. Graham | Eureka, Cal. | D. H. Strickland | Erie, Pa. |
| W. M. Griffiths | Louisville, Ky. | Jos. Taylor | Shreveport, La. |
| G. A. Harding | Sault Ste. Marie, Mich | W. H. Taylor | New Bedford, Mass. |
| W. H. Heard | Newport, Ark. | A. S. Tebbs | Marquette, Mich. |
| B. S. Herndon | Fredericksburg, Va. | J. H. Vandeman | Chattanooga, Tenn. |
| H. S. Hersey | Bismarck, Dak. | M. F. Wentworth | Portsmouth, N. H. |
| L. W. Hodgkins | Ellsworth, Me. | C. A. Wheaton | St Paul, Minn. |
| S. B. Hunter | Machias, Me. | R. C. White | Pensacola, Fla. |
| S. L. Jepson | Wheeling, W. Va. | Mortimer Wilson | Port Huron, Mich. |
| R. W. Johnson | Baltimore, Md. | J. E. Wood | Elizabeth City, N. C. |
| Sam'l Kitchen | East Saginaw, Mich. | H. S. Wyman | Sitka, Alaska. |
| T. J. McFarland | Indianola, Texas. | | |

